# Activation of the JAK/STAT3 and PI3K/AKT pathways are crucial for IL-6 trans-signaling-mediated pro-inflammatory response in human vascular endothelial cells

**DOI:** 10.1186/s12964-018-0268-4

**Published:** 2018-09-05

**Authors:** Mulugeta M. Zegeye, Madelene Lindkvist, Knut Fälker, Ashok K. Kumawat, Geena Paramel, Magnus Grenegård, Allan Sirsjö, Liza U. Ljungberg

**Affiliations:** 1 Cardiovascular Research Center, School of Medical Sciences, Örebro University Södra Grev Rosengatan 32, 703 62, Örebro, Sweden; 2 Present address: Department of Biochemistry and Molecular Biology, Faculty of Medicine, Dalhousie University, Dalhousie Medicine New Brunswick, Saint John, NB E2L 4L5 Canada

**Keywords:** Interleukin-6 signaling, Monocyte chemoattractant Protein-1, Pro-inflammatory cytokines, Endothelium, HUVECs

## Abstract

**Background:**

IL-6 classic signaling is linked to anti-inflammatory functions while the trans-signaling is associated with pro-inflammatory responses. Classic signaling is induced via membrane-bound IL-6 receptor (IL-6R) whereas trans-signaling requires prior binding of IL-6 to the soluble IL-6R. In both cases, association with the signal transducing gp130 receptor is compulsory. However, differences in the downstream signaling mechanisms of IL-6 classic- versus trans-signaling remains largely elusive.

**Methods:**

In this study, we used flow cytometry, quantitative PCR, ELISA and immuno-blotting techniques to investigate IL-6 classic and trans-signaling mechanisms in Human Umbilical Vein Endothelial Cells (HUVECs).

**Results:**

We show that both IL-6R and gp130 are expressed on the surface of human vascular endothelial cells, and that the expression is affected by pro-inflammatory stimuli. In contrast to IL-6 classic signaling, IL-6 trans-signaling induces the release of the pro-inflammatory chemokine Monocyte Chemoattractant Protein-1 (MCP-1) from human vascular endothelial cells. In addition, we reveal that the classic signaling induces activation of the JAK/STAT3 pathway while trans-signaling also activates the PI3K/AKT and the MEK/ERK pathways. Furthermore, we demonstrate that MCP-1 induction by IL-6 trans-signaling requires simultaneous activation of the JAK/STAT3 and PI3K/AKT pathways.

**Conclusions:**

Collectively, our study reports molecular differences in IL-6 classic- and trans-signaling in human vascular endothelial cells; and elucidates the pathways which mediate MCP-1 induction by IL-6 trans-signaling.

**Electronic supplementary material:**

The online version of this article (10.1186/s12964-018-0268-4) contains supplementary material, which is available to authorized users.

## Background

Vascular endothelial cells are constantly exposed to numerous types of circulating signaling molecules; and are one of the main targets for various pro- and anti-inflammatory cytokines [1]. Interleukin-6 (IL-6) is a multifunctional cytokine produced by several cell types including monocytes/macrophages, adipocytes, hematopoietic and endothelial cells [2]. IL-6 is known to regulate the synthesis of acute phase proteins and is elevated in the circulation during inflammatory conditions [2]. Furthermore, IL-6 has been shown to induce the expression of endothelial adhesion molecules and chemoattractant proteins such as the Monocyte Chemoattractant Protein-1 (MCP-1) [3, 4]. MCP-1 (also called CCL-2) is a potent monocyte chemoattractant that is secreted by numerous types of cells including endothelial cells and contributes to the initiation and progression of atherosclerotic plaques [5–7].

Numerous clinical trials using anti-IL-6 antibodies or antibodies directed against the IL-6 receptor (IL-6R) have shown therapeutic significance of blocking IL-6 signaling in chronic inflammatory diseases, including atherosclerosis [8, 9]. However, such systemic blockade of IL-6 has also been associated with adverse effects including severe infections [10, 11]. In addition, there are studies reporting a regenerative, athero-protective and anti-inflammatory role for IL-6 [12–14]. These dual roles of IL-6 in inflammation appears to originate from how IL-6 interacts with the specific target cell. IL-6 can act on target cells by binding to the membrane-bound receptor (IL-6R) and subsequent recruitment of ubiquitously expressed signal transducing gp130 receptor. This is known as IL-6 classic signaling and it is restricted to cells possessing membrane-bound IL-6R including hepatocytes, macrophages, neutrophils and some T cell subsets [15]. These cells also release the soluble receptor (sIL-6R) into the circulation predominantly through extracellular shedding or by alternative splicing [16–18]. Hence, IL-6 can alternatively bind to sIL-6R and induce intra-cellular signaling via gp130 on cells that lack the membrane-bound IL-6R, which is referred to as IL-6 trans-signaling [19, 20]. The trans-signaling is associated with pro-inflammatory functions while the classic signaling is linked to regenerative and anti-inflammatory functions [21, 22]. However, little is known about the signaling mechanisms of IL-6 classic- and trans-signaling. In this study we elucidated differences in classic- versus trans-signaling of IL-6 in human vascular endothelial cells and revealed a novel pathway by which IL-6 trans-signaling mediates its pro-inflammatory effect.

## Methods

### Cell culturing

Human Umbilical Vein Endothelial Cells (HUVECs, Life technologies, USA), were cultured in 75cm^2^ flasks (Sarstedt, Germany) containing complete endothelial medium [VascuLife basal medium supplemented with VEGF LifeFactors kit (LifeLine Cell Technologies, USA)] and antibiotics [Penicillin (0.1 U/ml) + Streptomycin (100 ng/ml)-PEST, Gibco, Life Technologies, USA]. The cultures were kept at 37 °C and 5% CO_2_ environment and cells were maintained until passage 10 by replacing medium every 48–72 h and/or sub-culturing upon confluence.

### Treatment of HUVECs

HUVECs (3 × 10^5^ cells/well for 6-well plates and 6 × 10^4^ cells/well for 24-well plates) were plated overnight in complete endothelial medium containing antibiotics. The next day, the medium was replaced with fresh antibiotics free medium and cells were treated with different concentrations of IL-6, IL-6R, TNF-α and LPS (all from R&D systems, USA) and/or pharmacological inhibitors CP690550, Stattic and LY294002 (all from R&D systems, USA), and PD98059 (Santa Cruz biotechnology, USA) for different time points ranging from 5 min to 48 h. At the end of incubations, supernatants and cells were collected and kept at -80 °C until further analysis. The cells were used to extract total protein or RNA.

### Gene knockdown

HUVECs were seeded in 6-well plates (2 × 10^5^ cells/well) containing complete endothelial medium with antibiotics and incubated overnight. After washing the cells with opti-MEM (Gibco, Life Technologies, USA), incubation continued with 700 μl opti-MEM/well containing 4 μl lipofectamine (Invitrogen, USA) and a mix of 3 stealth siRNAs (10 nM of each siRNA, Invitrogen, USA) targeting same gene. The control wells instead had non-target siRNAs (30 nM, Invitrogen, USA). After 4 h of incubation, 1.3 ml of complete endothelial medium was added into each well and incubation continued. At 48 h, culture supernatants and cells were collected and kept at -80 °C until further analysis.

### Total RNA isolation and cDNA synthesis

Total RNA extraction from frozen cells was achieved using E.Z.N.A® Total RNA Kit (OMEGA bio-tek inc, USA) according to manufacturer’s instructions. Briefly, TRK lysis buffer with 2% β-Mercaptoethanol was added into each well and the lysates were then mixed with 70% ethanol. The cell lysates were transferred into HiBind RNA columns and centrifuged for 1 min at 10,000 g. After washing the columns three times, RNA was eluted using RNase free water. RNA concentrations were determined using NanoDrop™ 2000 (Thermo Fisher Scientific, USA) spectrophotometer.

Extracted RNA were used to synthesize cDNA using high capacity cDNA reverse transcription kit (Thermo Fisher Scientific, USA) according to manufacturer’s instructions. Hence, 1 μg RNA extract was mixed with master mix composed of buffer, random primers, dNTPs and reverse transcriptase enzyme. The total reaction volume was adjusted to 20 μl by adding nuclease free water. A negative control containing master mix and water instead of RNA was also included. The following setup was used for thermal cycling: 10 min at 25 °C, 120 min at 37 °C, 5 min at 85 °C and kept at 4 °C before storage at − 20 °C.

### Real-time PCR

mRNA expression of genes was studied using TaqMan qPCR primers/probes according to manufacturer’s instructions. In short, a standard curve was prepared by pooling equal volume of cDNA from each sample that was then serially diluted in to 6 standards. Standards, negative controls and unknown samples were run in duplicate in a 96-well PCR plate. The total reaction volume was 10 μl consisting of LuminoCt qPCR ready mix (Sigma-Aldrich, USA), TaqMan Primer/Probe (Applied Biosystems, Life technologies, USA), water and cDNA. The cycling condition used was as follows: at 95 °C for 1 s and at 60 °C for 20 s for 40 cycles in addition to one step initialization at 95 °C for 20 s in ABI 7900HT Fast Real-Time PCR system (Applied Biosystems). Then, relative quantities were recorded for each well and normalized to the expression of housekeeping gene, GAPDH.

### Sandwich enzyme linked Immuno-sorbent assay (ELISA)

DuoSet® ELISA kits (R&D systems, USA) were used according to manufacturer’s instruction to determine the release of MCP-1 from HUVECs and expression of the IL-6-receptor and gp130 in HUVEC culture supernatants and cell lysates. Multiskan-Ascent (Lab Systems, Thermo Fisher Scientific, USA) Spectrophotometer was used to read absorbance at 450 nm. For IL-6R ELISA, 2 more standards to the lower end were included to cover low concentrations in samples.

### Protein extraction and quantification

HUVECs were rinsed with PBS and lysed using ice-cold RIPA lysis buffer (Millipore, USA). To quantify the proteins, Micro BCA™ Protein Assay kit was used (Thermo Scientific, USA) according to manufacturer’s instructions and absorbance at 540 nm was measured using Multiskan-Ascent (Lab Systems, Thermo Fisher Scientific, USA) Spectrophotometer.

### Immuno-(western) blotting

Cell lysates were mixed with 4× SDS sample buffer and denatured for 5 min at 95 °C. Next, the mixture (10 μg protein/well) was loaded on to 4–12% NuPAGE® Novex Bis-Tris gels and protein separation was achieved with MOPS/MES running buffers (both Invitrogen, USA). To determine molecular masses of the proteins, MagicMark™ XP Western Protein Standard (Invitrogen, USA) was used. Proteins were blotted onto Immobilon-FL PVDF membranes (Millipore, USA). For further steps TBS-T (10 mM Tris-HCl pH 8.0, 150 mM NaCl, 0.1% (*w*/*v*) Tween-20) was used. For detecting signaling proteins, membranes were incubated with the following primary antibodies: anti-phospho-Stat3^Tyr705^ antibody (Cell Signaling Technology, USA, #9131; 1:1000 dilution), anti-phospho-AKT^Ser473^ antibody (Cell Signaling Technology, USA, #4060; 1:2000 dilution), anti-phospho-ERK1/2 antibody (Cell Signaling Technology, USA, #9106; 1:2000 dilution), anti-STAT3 antibody (Cell Signaling Technology, USA, #4904; 1:2000 dilution), anti-AKT antibody (Cell Signaling Technology, USA, #2920; 1:2000 dilution), anti-ERK1/2 antibody (Cell Signaling Technology, USA, #4695; 1:1000 dilution), anti-phospho IκBα^Ser32^ antibody (Cell Signaling Technology, USA, #2859; 1:1000 dilution), anti-phospho-NFκB p65^Ser536^ antibody (Cell Signaling Technology, USA, #3033; 1:1000 dilution), anti-NFκB p65 antibody (Cell Signaling Technology, USA, #6956; 1:1000 dilution), anti-IκBα antibody (Santa Cruz Biotechnology, USA, SC-371; 1:750 dilution), anti-p52 antibody (Millipore, USA, #05–361; 1:1000 dilution), and anti-β-Tubulin antibody (Millipore, USA, #05–661; 1:2000 dilution). This was followed by incubation with horseradish peroxidase (HRP)-conjugated goat anti-rabbit IgGs (Cell Signaling Technology, USA, #7074; 1:2000) or horse anti-mouse IgGs (Cell Signaling Technology, USA, #7076; 1:2000). Protein bands were visualized using Immobilon™ Western Chemiluniescent HRP Substrate solution from Millipore (Millipore, USA), and chemiluminiscence was recorded by a Li-Cor Odyssey Fc imager and analyzed with Image Studio Software (both Li-Cor Biotechnology UK Ltd., United Kingdom). Stripping of membranes for re-probing was done according to manufacturer’s instructions using Restore™ Plus western blot stripping buffer (Thermo Fisher Scientific, USA, #46430).

### Flow cytometry

EDTA detached HUVECs were washed twice with PBS containing 1 mM EDTA and 2% FBS; and then stained with α-CD130 (gp130)-APC (clone: 2E1B02) and α-CD126 (IL-6Rα)-PE (clone: UV4) antibodies (BioLegend, UK) for 25 min at 4 °C in the dark. Fluorescence minus one (FMO) controls were used as negative control for staining. 7AAD staining was performed to remove dead cells from analyses. Stained cells were acquired using Gallios™ Flow Cytometer (Beckman Coulter Life Sciences, UK) and analyzed using Kaluza flow cytometry analysis software version 1.3 (Beckman Coulter, UK).

### Statistical analysis

Data were analyzed using GraphPad Prism® statistical software version 5.0 (GraphPad Software, Inc., USA). Data are presented as mean ± standard error of the mean (SEM) of at least 3 sets of experiments. For comparison between groups, paired t-test/Wilcoxon matched paired test and one-way ANOVA for repeated measures followed by Bonferroni post-hoc test was used. *p* value less than 0.05 was considered as statistically significant.

## Results

### Expression and regulation of IL-6 receptors in vascular endothelial cells

**Fig. 1:**
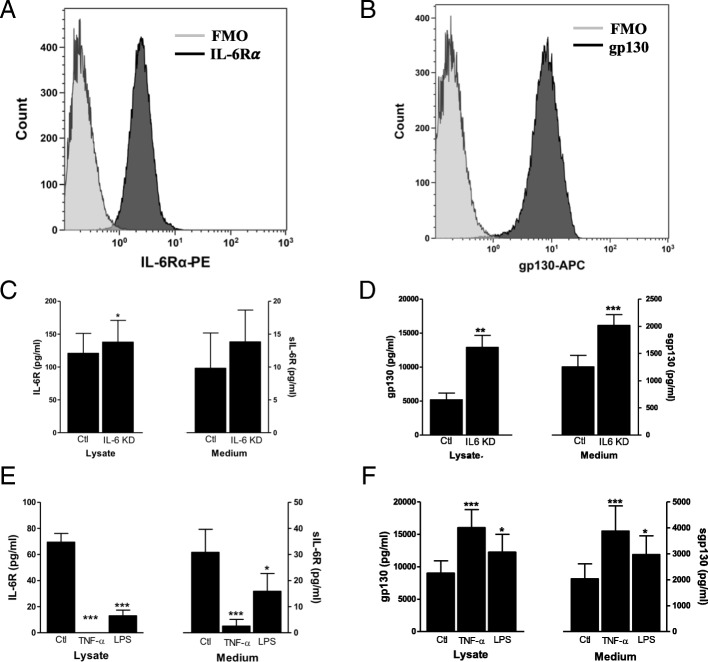
Expression and regulation of IL-6R and gp130 in human vascular endothelial cells. Flow cytometry analyses of EDTA detached endothelial cells stained for **a** IL-6R and **b** gp130. The light grey histograms show respective FMO (fluorescence minus one) controls and the dark grey histograms show IL-6R or gp130 stained cells. ELISA analyses of the levels of **c** IL-6R and **d** gp130 in cell lysates and medium after 48 h of IL-6 knockdown. ELISA analyses of the levels of **e** IL-6R and **f** gp130 in cell lysates and medium after 48 h of treatment with TNF-α (50 ng/ml) and LPS (100 ng/ml). Data is presented as mean ± SEM of at least 3–4 experiments each run-in duplicate. *p < 0.05, **p < 0.01, ***p < 0.001 compared to control

To study whether human vascular endothelial cells express IL-6R and gp130, we used flow cytometry to detect their surface protein expression. As depicted in Fig. 1a & b, both IL-6R and gp130 proteins are expressed on surface of human vascular endothelial cells. We also assessed how different pro-inflammatory stimuli could regulate expression of IL-6R and gp130 in these cells using ELISA. Knockdown of IL-6 (80–90% knockdown efficiency, Additional file 1: Figure S1) increased the levels of IL-6R and gp130 (Fig. 1c & d). In addition, treatment of endothelial cells with pro-inflammatory stimuli (TNF-α or LPS) resulted in downregulation of both IL-6R and sIL-6R while upregulating gp130 and sgp130 levels (Fig. 1e & f). Overall, these findings demonstrate that IL-6R and gp130 are expressed on human vascular endothelial cells, allowing for the activation of both IL-6 classic- and trans-signaling in these cells.

### MCP-1 induction in human vascular endothelial cells in response to IL-6

**Fig. 2:**
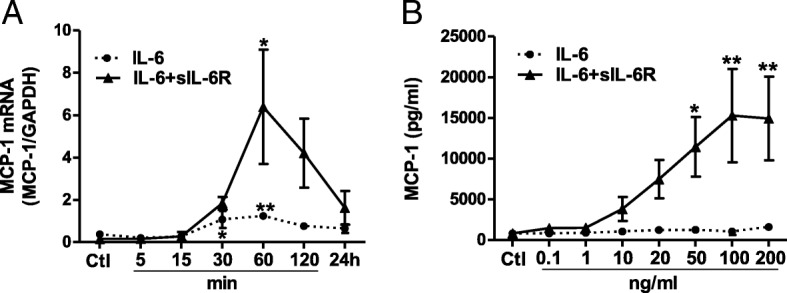
MCP-1 mRNA expression and release from human vascular endothelial cells. **a** qPCR analyses depicting the relative expression of MCP-1 mRNA after exposure to IL-6 (100 ng/ml) alone or in combination with sIL-6R (200 ng/ml). **b** ELISA data showing the MCP-1 release into medium (48 h) induced by increasing concentrations of IL-6 alone or in combination with sIL-6R. Data is presented as mean ± SEM of 3 experiments each run-in duplicate. *p < 0.05, **p < 0.01 compared to its respective control

Next, we investigated the response of human vascular endothelial cells to the treatment with IL-6 alone or in combination with sIL-6R on the expression and release of MCP-1. We found that IL-6 in combination with sIL-6R induced a prominent increase in MCP-1 mRNA that peaked after 60 min, while IL-6 alone had a very limited effect (Fig. 2a). In addition, we show that stimulation with IL-6 in combination with sIL-6R, but not IL-6 alone, induced a dose-dependent release of MCP-1 into the medium (Fig. 2b). Further, stimulation of human vascular endothelial cells with combination of IL-6 and sIL-6R, but not IL-6 alone, upregulated cell adhesion molecules VCAM-1 and ICAM-1 (Additional file 2: Figure S2A and 2B). These findings indicate that IL-6 requires the sIL-6R to induce pro-inflammatory responses in human vascular endothelial cells.

### Activation of signaling pathways by IL-6 with or without sIL-6R

**Fig. 3:**
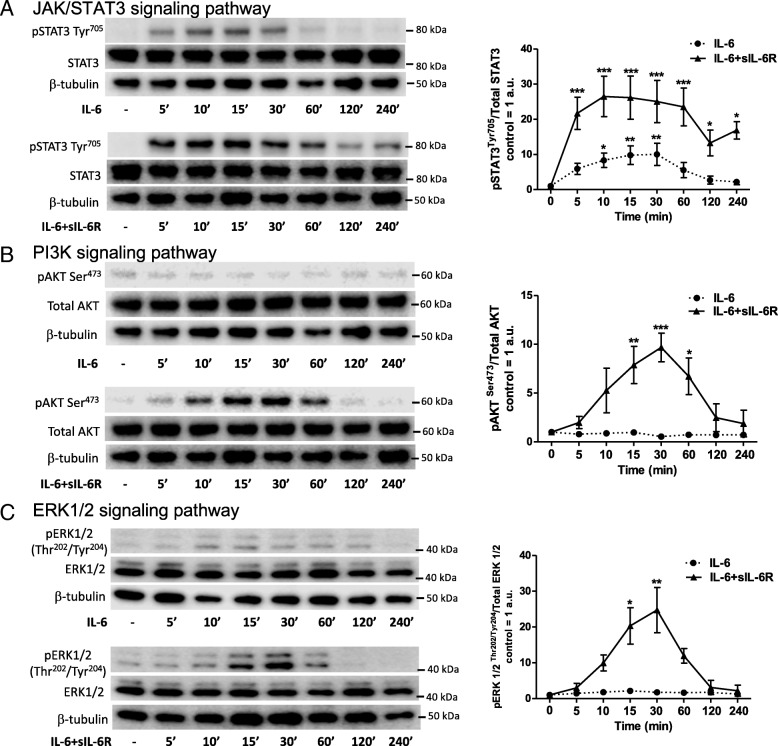
Western blot analyses showing phosphorylation of **a** STAT3^Tyr705^, **b** AKT^Ser473^ and **c** ERK1/2^Thr202/Tyr204^ in human vascular endothelial cells treated with IL-6 alone (50 ng/ml) or in combination with sIL-6R (100 ng/ml). One representative blot containing the phosphorylated protein, total protein and β-tubulin (loading control) is shown for each pathway (left column). The signals from the phosphorylated proteins and total proteins are first normalized to β-tubulin, and the ratio of the phosphorylated proteins and the total proteins are calculated. The graphs show arbitrary units (a.u., control is set to 1) compiled from 3 independent experiments presented as mean ± SEM for each pathway (right column). *p < 0.05, **p < 0.01, ***p < 0.001 compared to control

In order to investigate the different signaling pathways engaged, we treated human vascular endothelial cells with IL-6 alone (classic signaling) or together with sIL-6R (trans-signaling) for increasing durations. We then analyzed activation of different signaling pathways using immuno-(western) blotting. We found that phosphorylation of STAT3^Tyr705^ is induced by both IL-6 alone and in combination with sIL-6R (Fig. 3a, left) in a dose-dependent manner (Additional file 3: Figure S3). When applied together with sIL-6R, very low concentrations of IL-6 (i.e. 1 ng/ml) were sufficient to induce STAT3^Tyr705^ phosphorylation (Additional file 3: Figure S3), while higher concentrations (i.e. 50 ng/ml) were required to induce similar degree of phosphorylation when IL-6 is added without sIL-6R. Although IL-6 in combination with sIL-6R provoked a stronger phosphorylation of STAT3^Tyr705^ compared to IL-6 alone, both treatments follow similar kinetics in which the phosphorylation peaked after 10-15 min of stimulation (Fig. 3a, right).

In addition, we found that IL-6 alone does not induce phosphorylation of AKT^Ser473^ (Fig. 3b, left) or ERK1/2^Thr202/Tyr204^ (Fig. 3c, left). However, when applied in combination with sIL-6R, IL-6 causes a strong AKT^Ser473^ (Fig. 3b, right) as well as ERK1/2^Thr202/Tyr204^ (Fig. 3c, left) phosphorylation which peaked at 30 min after treatment. We also investigated whether IL-6 induces activation of NFκB signaling pathways by determining the level of p-p65^Ser32^, pIκB^Ser536^ and IκB for canonical NFκB activation; and the level of p100 and p52 for non-canonical NFκB activation. The treatment of human vascular endothelial cells with IL-6 alone or in combination with sIL-6R did not cause phosphorylation of p65^Ser32^ or IκB^Ser536^ nor did it reduce the level of IκB (Additional file 4: Figure S4), unlike the case for TNF-α stimulation (data not shown). Moreover, the levels of p100 and p52 were not affected by neither IL-6 alone nor in combination with sIL-6R (Additional file 4: Figure S4). These findings indicate that IL-6 signaling pathways are distinct; in which classic signaling engages the JAK/STAT3 pathway while trans-signaling employs the JAK/STAT3, PI3K/AKT, and the MEK/ERK signaling pathways. In addition, neither canonical- nor non-canonical NFκB signaling pathways seem to be activated by either IL-6 classic or trans-signaling.

### Pathways mediating IL-6 trans-signaling induced MCP-1 in human vascular endothelial cells

**Fig. 4:**
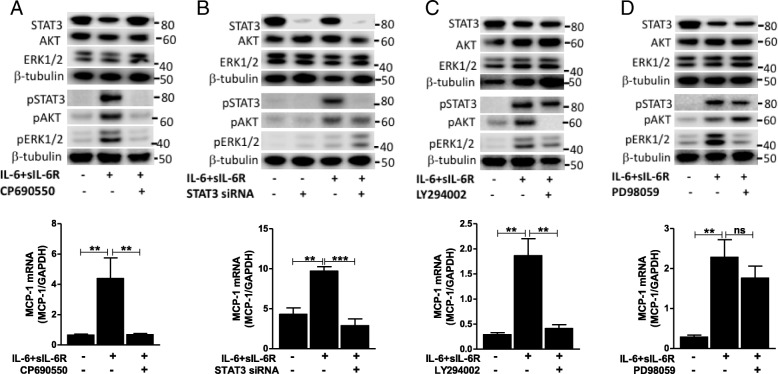
The effect of different inhibitions on IL-6 trans-signaling induced MCP-1 mRNA expression. The effect of **a** CP960550 (JAK inhibitor, 10 μM), **b** STAT3 knockdown, **c** LY294002 (PI3K inhibitor, 50 μM) and **d** PD98059 (MEK inhibitor, 10 μM) on trans-signaling (IL-6 = 100 ng/ml, sIL-6R = 200 ng/ml) induced MCP-1 expression is presented as mean ± SEM (*n* = 3 for each). Western blots show effect of CP960550, STAT3 knockdown, LY294002 and PD98059 on IL-6 trans-signaling (IL-6 = 50 ng/ml, sIL-6R = 100 ng/ml) induced phosphorylation of downstream pathway proteins. *p < 0.05, **p < 0.01, ***p < 0.001 compared to control

To further elucidate the pathways accounting for the regulation of MCP-1 by IL-6 trans-signaling, we used pharmacological inhibitors or siRNA to interfere with the different pathways and assessed MCP-1 expression after exposure to IL-6 in combination with sIL-6R. We found that pre-treatment of human vascular endothelial cells with the JAK inhibitor CP690550 prevents the trans-signaling induced phosphorylation of STAT3^Tyr705^, AKT^Ser473^ and ERK1/2^Thr202/Tyr204^ (Fig. 4a). In addition, CP690550 pre-treatment downregulated the MCP-1 expression evoked via trans-signaling in these cells (Fig. 4a). These findings indicate that JAK is upstream of STAT3, AKT and ERK1/2 in the signaling pathway induced by IL-6 trans-signaling, and that JAK is crucial for the induction of MCP-1.

Knockdown of STAT3 in human vascular endothelial cells using siRNA (knockdown efficiency 80–90%) abolished the phosphorylation of STAT3^Tyr705^ caused by IL-6 trans-signaling (Fig. 4b). In line with that, qPCR analyses revealed that MCP-1 induction by IL-6 trans-signaling was eliminated in STAT3 knockdown cells (Fig. 4b). Similarly, treating human vascular endothelial cells with the PI3K inhibitor LY294002 prior to stimulation with IL-6 combined with sIL-6R resulted in the suppression of AKT^Ser473^ phosphorylation (Fig. 4c). Furthermore, MCP-1 induction by IL-6 trans-signaling was inhibited by LY294002 pre-treatment (Fig. 4c). Pre-treatment with the MEK inhibitor PD98059 blocked IL-6 trans-signaling induced phosphorylation of ERK1/2^Thr202/Tyr204^(Fig. 4d), but not MCP-1 expression in human vascular endothelial cells (Fig. 4d). Overall, our findings demonstrate that IL-6 trans-signaling employs the JAK/STAT3 and PI3K/AKT pathways to provoke MCP-1 expression in human vascular endothelial cells.

## Discussion

In this study, we elucidated differences in classic- versus trans-signaling of IL-6 in human vascular endothelial cells. Our results show that human vascular endothelial cells express IL-6R and gp130 on their surface allowing for the activation of both IL-6 classic- and trans-signaling in these cells. We demonstrate that IL-6 classic signaling causes transient phosphorylation of STAT3^Tyr705^, while trans-signaling also leads to phosphorylation of AKT^Ser473^ and ERK1/2^Thr202/Tyr204^. Further, we report that IL-6 trans-signaling induces the expression and release of the pro-inflammatory chemokine MCP-1 in human vascular endothelial cells, and that this is mediated via JAK/STAT3 and PI3K/AKT pathways.

The general assumption is that the expression of IL-6R is restricted to only a few cell types, such as hepatocytes and immune cells. However, we provide evidence that IL-6R is expressed on the surface of human vascular endothelial cells. This finding is in contrast to previous studies which could not to determine IL-6R expression on these cells [23], and indirect implications from the lack in responsiveness of the cells to IL-6 [24]. We also show that vascular endothelial cells release IL-6R in its soluble form, and that its expression is regulated by pro-inflammatory agents. In agreement with findings in monocytes, TNF-α [25] and LPS [26] treatment repress the level of both membrane-bound and soluble IL-6R in vascular endothelial cells. On the other hand, both TNF-α and LPS upregulate gp130 surface expression and its release from vascular endothelial cells. This indicates that pro-inflammatory stimuli render endothelial cells to become more responsive to trans-signaling and may favor a pro-inflammatory response. However, it is important to note that gp130 is a common signal transducing receptor for several cytokines and might also initiate other responses. Our results show that activation of classic signaling induces a minor induction of MCP-1 mRNA although it does not affect the protein release. The trans-signaling, however, induces a prominent increase in MCP-1 mRNA which corresponds to significantly higher level of MCP-1 release. These latter findings are consistent with previous reports [23, 24].

The IL-6R has only a short intracellular domain and its signal transduction is based on ligand-binding dependent recruitment of gp130 to which JAKs (JAK1, JAK2 and TYK2) are closely associated [27, 28]. Hence, IL-6 is generally known to activate JAK/STAT3 pathway regardless of binding to soluble or membrane bound IL-6R. In line with this, we show that both classic- and trans-signaling of IL-6 trigger STAT3^Tyr705^ phosphorylation in a dose- and time-dependent manner. However, the trans-signaling causes a markedly increased STAT3^Tyr705^ phosphorylation compared to classic signaling. Furthermore, we report that IL-6 trans-signaling, but not classic signaling, leads to an activation of the PI3K/AKT and MEK/ERK pathways. It has previously been suggested that gp130-mediated activation of these pathways is achieved by the recruitment of SHP-2 (SH2 domain-containing protein-tyrosine phosphatase) to intracellular domain of gp130 (Tyr^759^) [29]. The same phospho-site also serves as docking site for SOCS3 (suppressor of cytokine signaling 3), a key negative regulator of IL-6 signaling pathways [30]. MCP-1 production by human vascular endothelial cells in response to pro-inflammatory mediators has been shown to involve NFκB pathways [31]. Moreover, IL-6 has been shown to trigger NFκB activation in intestinal epithelial cells thereby promoting inflammation [32]. We found that neither IL-6 classic- nor trans-signaling induce activation of the canonical or non-canonical NFκB pathways, indicating that the induction of MCP-1 by IL-6 trans-signaling is independent of NFκB pathways in vascular endothelial cells.

The involvement of STAT3 in IL-6 trans-signaling induced MCP-1 production from vascular endothelial cells has been previously reported by the use of STAT3 inhibitors such as Stattic [24]. In our hands, however, this inhibitor strongly affected cell viability (data not shown) and therefore we employed siRNA guided STAT3 knockdown instead. In line with previous findings [24], we determined that STAT3 is essential for the trans-signaling mediated MCP-1 expression in vascular endothelial cells. Nevertheless, blockade of PI3K/AKT pathway also resulted in a complete inhibition of trans-signaling caused AKT^Ser473^ phosphorylation as well as MCP-1 induction. This indicates that MCP-1 induction by IL-6 trans-signaling requires simultaneous activation of the JAK/STAT3 and PI3K/AKT pathways. It is well known that activation of the JAK/STAT3 pathway leads to STAT3 dimerization and translocation into the nucleus where it initiates gene transcription [33]. However, the downstream effects of PI3K signaling are mostly associated with activation of S6 kinases (e.g. S6 K1) and inhibition of 4E-binding protein 1 (4E-BP1), both participating in post-transcriptional regulation [34]. Given the significant reduction in MCP-1 mRNA expression by PI3K signaling inhibition, it is likely that activation of the PI3K pathway also activates downstream transcription factors. Several mechanisms by which PI3K regulates gene transcription have been shown previously [35–37]. Furthermore, we show that the blockade of the MEK/ERK pathway abolishes trans-signaling induced ERK1/2^Thr202/Tyr204^ phosphorylation while the MCP-1 induction remains unaffected. These findings suggest that ERK1/2 activation in response to IL-6 trans-signaling has a minor role in MCP-1 induction in human vascular endothelial cells. In contrast to our findings, the MEK/ERK pathway has been shown to be involved in IL-6 induced regulation of MCP-1 in fibroblasts [38]. This may be explained by a cell type specific and distinct activation of pathways and responses caused by IL-6 trans-signaling. As expected, inhibition of JAK abrogated trans-signaling induced phosphorylation of STAT3^Tyr705^, AKT^Ser473^, and ERK1/2^Thr202/Tyr204^ and abolished MCP-1 induction.

## Conclusions

Put together, this study sheds light on differences in IL-6 classic- and trans-signaling on molecular level in human vascular endothelial cells, demonstrating that concurrent activation of the JAK/STAT3 and PI3K/AKT pathways is essential for trans-signaling induced MCP-1 production.

## Additional files

Additional file 1: **Figure S1.** Bar graph showing ELISA analyses on IL6 release from human vascular endothelial cells during IL6 knockdown compared to control. Data is presented as mean ± SEM of 8 experiments each run-in duplicate. ***p < 0.01 compared to control. (PDF 14 kb)

Additional file 2: **Figure S2.** Bar graphs showing mRNA expression of cell adhesion molecule (A) VCAM-1 and (B) ICAM-1 by human vascular endothelial cells in response to stimulation with IL-6 and sIL-6R (24 h). Data is presented as mean ± SEM of 3 experiments each run-in duplicate. **p < 0.01, ***p < 0.001 compared to control. (PDF 19 kb)

Additional file 3: **Figure S3.** Phosphorylation of STAT3^Tyr705^ in response to increasing concentration of (A) IL-6 alone or (B) in combination with sIL-6R. One representative blot and total STAT3 (loading control) is shown (left column). The graphs show arbitrary units (a.u., control is set to 1) compiled from 2 to 3 independent experiments presented as mean ± SEM for each pathway (right column). *p < 0.05, **p < 0.01 compared to control. (PDF 235 kb)

Additional file 4: **Figure S4.** Western blot analyses showing the levels of pIκB^Ser32^/IκB and -p65^Ser32^/p65 (canonical NFκB), and p100/p52 (non-canonical NFκB) in vascular endothelial cells stimulated with IL-6 alone (50 ng/ml, top section) or in combination with sIL-6R (100 ng/ml, bottom section). (PDF 177 kb)

## Abbreviations

a.u: Arbitrary units
FMO: Fluorescence minus one
gp130: Glyco-protein-130
HUVECs: Human Umbilical Vein Endothelial Cells
ICAM-1: Intercellular adhesion molecule-1
IL-6: Interleukin-6
IL-6R: IL-6 Receptor
MCP-1: Monocyte Chemo-attractant Protein-1
sgp130: Soluble-gp130
SHP-2: SH2 domain-containing protein-tyrosine phosphatase
sIL-6R: Soluble-IL-6R
SOCS3: Suppressor of Cytokine Signaling-3
VCAM-1: Vascular cell adhesion molecule-1
