# Supplementary figures and images for: Activation of the JAK/STAT3 and PI3K/AKT pathways are crucial for IL-6 trans-signaling-mediated pro-inflammatory response in human vascular endothelial cells
Source: Cell Commun Signal. 2018 Sep 5;16:55. doi: 10.1186/s12964-018-0268-4 (PMC6125866; doi:10.1186/s12964-018-0268-4)

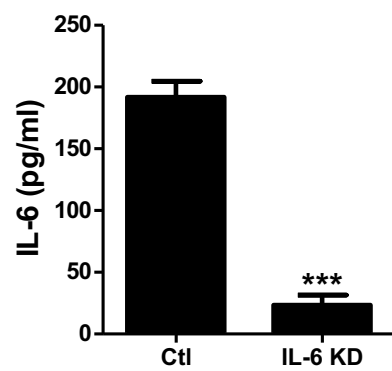

Supplement: Supplementary file 1 — Figure S1. Bar graph showing ELISA analyses on IL6 release from human vascular endothelial cells during IL6 knockdown compared to control. Data is presented as mean ± SEM of 8 experiments each run-in duplicate. ***p < 0.01 compared to control. (PDF 14 kb) [file 12964_2018_268_MOESM1_ESM.pdf]

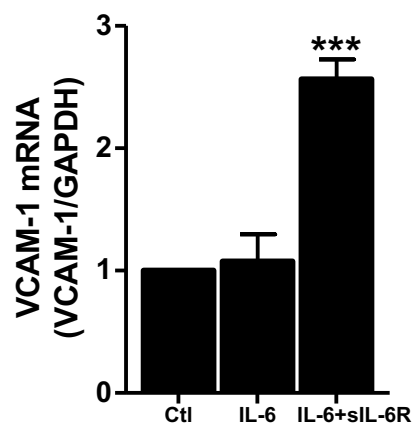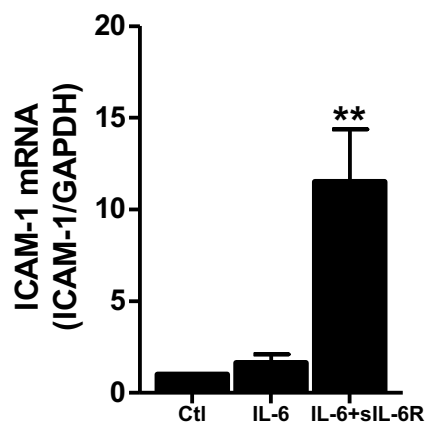

Supplement: Supplementary file 2 — Figure S2. Bar graphs showing mRNA expression of cell adhesion molecule (A) VCAM-1 and (B) ICAM-1 by human vascular endothelial cells in response to stimulation with IL-6 and sIL-6R (24 h). Data is presented as mean ± SEM of 3 experiments each run-in duplicate. **p < 0.01, ***p < 0.001 compared to control. (PDF 19 kb) [file 12964_2018_268_MOESM2_ESM.pdf]

A)

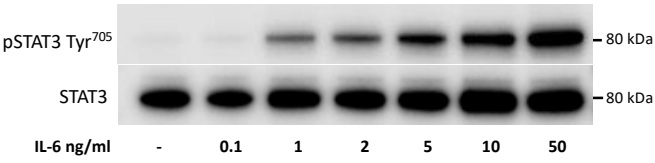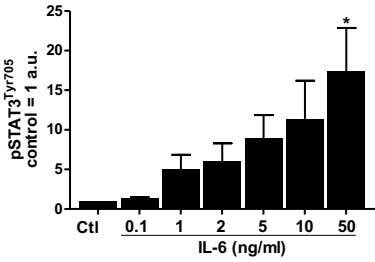

B)

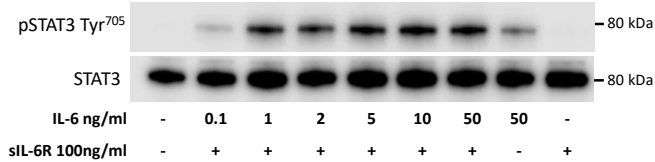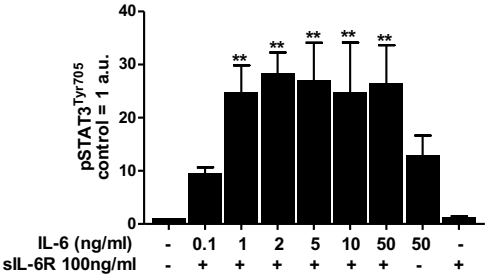

Supplement: Supplementary file 3 — Figure S3. Phosphorylation of STAT3Tyr705 in response to increasing concentration of (A) IL-6 alone or (B) in combination with sIL-6R. One representative blot and total STAT3 (loading control) is shown (left column). The graphs show arbitrary units (a.u., control is set to 1) compiled from 2 to 3 independent experiments presented as mean ± SEM for each pathway (right column). *p < 0.05, **p < 0.01 compared to control. (PDF 235 kb) [file 12964_2018_268_MOESM3_ESM.pdf]
