# Supplementary material for: Activation of the JAK/STAT3 and PI3K/AKT pathways are crucial for IL-6 trans-signaling-mediated pro-inflammatory response in human vascular endothelial cells
Source: Cell Commun Signal. 2018 Sep 5;16:55. doi: 10.1186/s12964-018-0268-4 (PMC6125866; doi:10.1186/s12964-018-0268-4)
Supplement: Supplementary file 4 — Figure S4. Western blot analyses showing the levels of pIκBSer32/IκB and -p65Ser32/p65 (canonical NFκB), and p100/p52 (non-canonical NFκB) in vascular endothelial cells stimulated with IL-6 alone (50 ng/ml, top section) or in combination with sIL-6R (100 ng/ml, bottom section). (PDF 177 kb) [file 12964_2018_268_MOESM4_ESM.pdf]

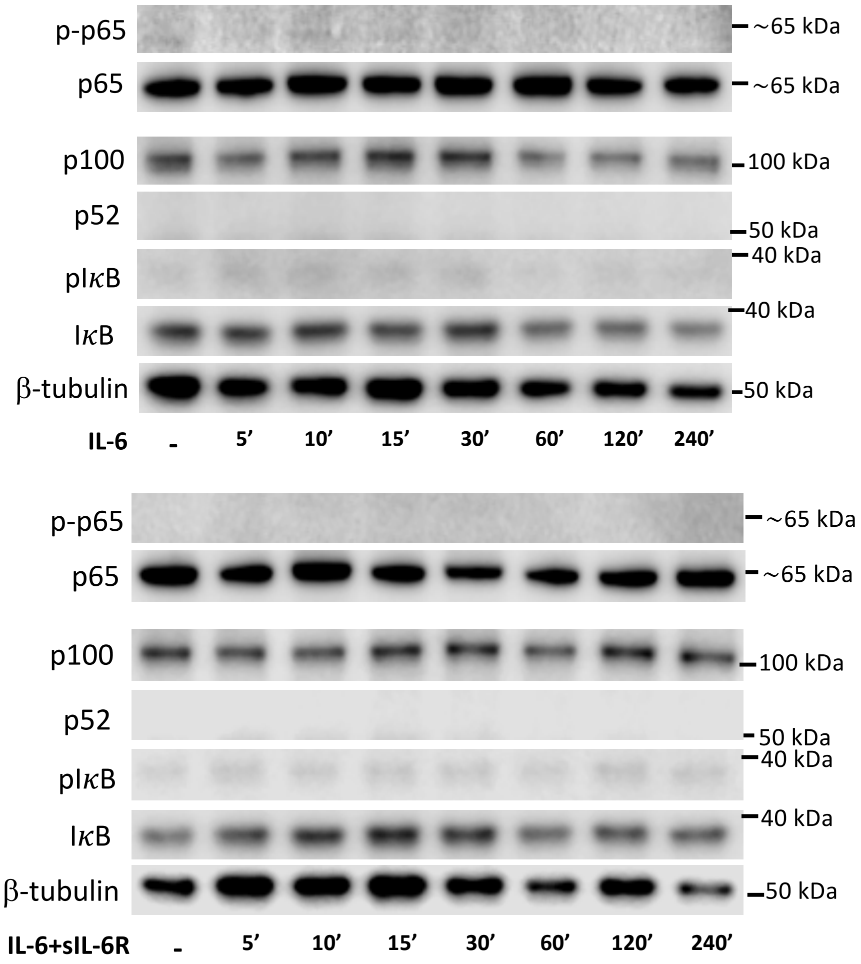

p-p65
~65 kDa
p65
~65 kDa
p100
100 kDa
p52
50 kDa
pIκB
40 kDa
IκB
40 kDa
β-tubulin
50 kDa
IL-6
-
5'
10'
15'
30'
60'
120'
240'
p-p65
~65 kDa
p65
~65 kDa
p100
100 kDa
p52
50 kDa
pIκB
40 kDa
IκB
40 kDa
β-tubulin
50 kDa
IL-6+sIL-6R
-
5'
10'
15'
30'
60'
120'
240'
